# High-Frequency Rugose Exopolysaccharide Production by *Vibrio cholerae* Strains Isolated in Haiti

**DOI:** 10.1371/journal.pone.0112853

**Published:** 2014-11-12

**Authors:** Mustafizur Rahman, Mohammad Jubair, Meer T. Alam, Thomas A. Weppelmann, Taj Azarian, Marco Salemi, Ilya A. Sakharuk, Mohammed H. Rashid, Judith A. Johnson, Mahmuda Yasmin, J. Glenn Morris, Afsar Ali

**Affiliations:** 1 Emerging Pathogens Institute, University of Florida, Gainesville, Florida, United States of America; 2 Department of Environmental and Global Health, College of Public Health and Health Professions, University of Florida, Gainesville, Florida, United States of America; 3 Department of Epidemiology, College of Public Health and Health Professions, University of Florida, Gainesville, Florida, United States of America; 4 Department of Pathology, Immunology, and Laboratory Medicine, College of Medicine, University of Florida, Gainesville, Florida, United States of America; 5 Department of Microbiology, University of Dhaka, Dhaka, Bangladesh; Loyola University Medical Center, United States of America

## Abstract

In October, 2010, epidemic cholera was reported for the first time in Haiti in over 100 years. Establishment of cholera endemicity in Haiti will be dependent in large part on the continued presence of toxigenic *V. cholerae* O1 in aquatic reservoirs. The rugose phenotype of *V. cholerae*, characterized by exopolysaccharide production that confers resistance to environmental stress, is a potential contributor to environmental persistence. Using a microbiologic medium promoting high-frequency conversion of smooth to rugose (S–R) phenotype, 80 (46.5%) of 172 *V. cholerae* strains isolated from clinical and environmental sources in Haiti were able to convert to a rugose phenotype. Toxigenic *V. cholerae* O1 strains isolated at the beginning of the epidemic (2010) were significantly less likely to shift to a rugose phenotype than clinical strains isolated in 2012/2013, or environmental strains. Frequency of rugose conversion was influenced by incubation temperature and time. Appearance of the biofilm produced by a Haitian clinical rugose strain (altered biotype El Tor HC16R) differed from that of a typical El Tor rugose strain (N16961R) by confocal microscopy. On whole-genome SNP analysis, there was no phylogenetic clustering of strains showing an ability to shift to a rugose phenotype. Our data confirm the ability of Haitian clinical (and environmental) strains to shift to a protective rugose phenotype, and suggest that factors such as temperature influence the frequency of transition to this phenotype.

## Introduction

Cholera continues to be a major public health threat globally particularly in countries where safe drinking water, adequate sanitation and hygiene are suboptimal [1]. Toxigenic *V. cholerae* strains within serogroups O1 and O139 are almost exclusively responsible for epidemic cholera; non-toxigenic strains outside of these O groups (non-O1/O139 *V. cholerae*) are widely distributed in the environment, but generally are not associated with human illness. *V. cholerae* has two lifestyles, including: (a) a transient passage through the human intestine where toxigenic strains can promotes profuse diarrhea (i.e. cholera), and (b) a ubiquitous existence in aquatic environments, including fresh, estuarine and marine environments [1], [2], [3]. In aquatic reservoirs, the microorganism can survive either in planktonic (free-living) form or in biofilms [2], [3]. Suggestions have been made that the bacteria survive between epidemics in these aquatic reservoirs, with environmental triggers causing seasonal increases in counts, followed by “spill-over” into human populations [1]. However, the genetic and physiologic basis of the persistence of *V. cholerae* in the environment, particularly during inter-epidemic period, is poorly understood.

In this context, three major suggestions have been made in regard to environmental persistence of *V. cholerae*. First, a body of literature suggests that *V. cholerae* can enter into a viable but non-culturable state (VBNC) in response to nutrient starvation and/or cold temperature [4], [5]. Second, as recently reported by our group, a subset of *V. cholerae* cells can assume a “persister” phenotype by stochastic mechanisms in response to nutrient-poor conditions [6]. Third, *V. cholerae* can reversibly switch from a smooth colony phenotype to a “rugose” (wrinkled) phenotype characterized by copious production of exopolysaccharide conferring resistance to chlorine, osmotic and oxidative stresses [7], [8], [9]. Exopolysaccharide secretion and biofilm production by many bacterial pathogens has been linked to efforts to evade stresses elicited by host immunity and by environmental pressures [10], [11], [12]. Studies involving *in vitro* conversion of smooth *V. cholerae* strains to the rugose variant have generally been limited to single laboratory isolates, in which a smooth isolate was exposed to stressful growth conditions to induce the conversion of the smooth phenotype to a rugose variant prior to plating the culture on agar [7], [9]. Generally the conversion of smooth cells to rugose variants (S to R conversion) has resulted in a conversion rate of 1–2% with incubation times ranged from several days to months [7], [9]. We have subsequently reported the use of a culture medium designated as “APW #3” which promotes high-frequency rugose exopolysaccharide production (as high as 60–80% rugose colonies) when a single colony of *V. cholerae* is incubated statically in that medium either at 30 or 37°C for 48–72 h prior to plating on L agar [8].

The 7^th^ pandemic *V. cholerae* O1 biotype El Tor strain, which first emerged in Indonesia in 1961 and ultimately replaced *V. cholerae* O1 classical biotype, has evolved to an “altered” *V. cholerae* O1 El Tor strain in early 2000. The altered *V. cholerae* O1 El Tor strain carries a classical *ctxB* gene and is associated with more severe disease. Despite the emergence of altered *V. cholerae* O1 El Tor strain and its subsequent spread and circulation in many parts of the world, including the recent cholera epidemic in Haiti [13], [14], no study has been undertaken to determine whether altered *V. cholerae* strains have rates of rugose exopolysaccharide production and biofilm formation comparable to those seen with typical *V. cholerae* O1 El Tor strains. Taking advantage of our ongoing clinical and environmental surveillance for *V. cholerae* strains in Haiti, we investigated the frequency with which circulating smooth altered *V. cholerae* strains in Haiti can switch to a rugose phenotype. Here we provide evidence that: (a) unlike typical *V. cholerae* O1 El Tor strains, the majority of the smooth phenotype of altered *V. cholerae* O1 strains isolated from Haiti switched to a rugose colony phenotype (S–R conversion) after at least 48 h of growth on L-agar following their growth in APW # 3 for 72 h; (b) in contrast to 2010 clinical O1 isolates, 2012 and 2013 clinical isolates from Haiti exhibited significantly increased S–R conversion; (c) S–R conversion was greatly influenced by incubation temperatures with cultures incubated at 37°C having significantly enhanced rugose production compared to those incubated at 25°C, and (d) the Haitian *V. cholerae* rugose colony phenotype was influenced by incubation temperatures.

## Materials and Methods

### Bacterial strains

A total of 172 *V. cholerae* strains isolated from Haiti were included in this study; numbers of isolates by serogroup (O1 vs. non-O1/O139), source (clinical vs. environmental), and year of isolation are shown in Table 1 and Table 2. Data on isolation and characteristics of these isolates, other than rugose production, have been previously reported [15]. A complete list of strains is included in Table S1; each isolate represents a single strain obtained from an independent clinical/environmental sample at a given collection/sampling time. The method used to isolate *V. cholerae* O1 and non-O1/non-O139 strains from water samples in Haiti was described in our recent report [15]. All the isolated strains were confirmed as *V. cholerae* using *V. cholerae* species-specific PCR assay as described previously [16]. Toxigenic *V. cholerae* O1 strains were further confirmed by serology and by the PCR amplification of virulence genes [16]. Canonical *V. cholerae* O1 biotype El Tor strains, N16961S (smooth) and its isogenic variant N16961R (rugose) and a smooth variant of *V. cholerae* O395 (serogroup O1 and biotype classical) were included as control strains. All the *V. cholerae* strains were stored in 30% glycerol at −80°C.

**Table 1:** Frequency of switching of smooth phenotype of *V. cholerae* strains to rugose phenotype.

Strains	Source	Year of isolation	Strains examined (n)	Conversion of smooth to rugose phenotype (%)
*V. cholerae* O1	Clinical	2010–13	70	30 (42.9)
*V. cholerae* O1	Environmental	2012–13	24	12 (50.0)
*V. cholerae* non-O1/non-O139	Environmental	2012–13	78	38 (48.7)
Total		2010–13	172	80 (46.5)

The strains were isolated from clinical and environmental sources in Haiti between 2010 and 2013.

**Table 2:** Conversion of smooth *V. cholerae* strains to rugose phenotype by years and sources of isolation.

Strains	Source	Year of isolation	Strains examined (n)	Conversion of smooth to rugose phenotype (%)
*V. cholerae* O1	Clinical	2010	14	1 (7.1)*
*V. cholerae* O1	Clinical	2012	27	18 (66.7)*
*V. cholerae* O1	Clinical	2013	29	11 (37.9)*
*V. cholerae* O1	Environmental	2012	7	5 (71.4)
*V. cholerae* O1	Environmental	2013	17	7 (41.2)
*V. cholerae* non-O1/non-O139	Environmental	2012	54	28 (51.9)
*V. cholerae* non-O1/non-O139	Environmental	2013	24	10 (41.7)
Total		2010–13	172	80 (46.5)

*Differences between 2010 and 2012, and 2010 and 2012 and 2013 combined, are statistically significant

### Screening for rugose colony phenotype and determining the percentage of rugose conversion by each isolate

To confirm that Haitian *V cholerae* strains can switch from a smooth to rugose colony phenotype, we used a previously described medium (APW #3) promoting high-frequency conversion of smooth phenotype *V. cholerae* to a rugose phenotype [8]. Briefly, each *V. cholerae* strain stored at −80°C was subcultured onto L-agar to obtain isolated colonies; a single smooth colony grown overnight at 37°C on L-agar was then inoculated into two 15 ml sterile glass tubes containing 3 ml APW# 3 broth. One culture tube was incubated overnight statically at 37°C for 72 h; the second culture tube was incubated overnight statically at 25°C for 72 h. After incubations, the culture tubes were vortexed vigorously for one minute to disrupt the aggregated cells, if any, and an appropriate dilution of each culture was then plated onto L-agar and the culture plate was incubated at 37°C for 18–24 h followed by incubation at room temperature for additional 48 h. Culture plates were examined each day to identify colonies having a rugose colony phenotype. A total of at least 150 colonies were examined for each strain at each experimental temperature. If a rugose colony was observed on any L-agar plate, the strain was considered to be a rugose-producing strain and the percentage of rugose conversion of that isolate was determined by dividing the total number of rugose colonies observed by the total colonies counted on the L-agar (Table 3). All the rugose-negative strains were reexamined for rugose production using methods described above.

**Table 3:** Effect of temperature on the conversion of smooth *V. cholerae* strains to the rugose phenotype.

Strains	Source	Year of isolation	Total number (n) of isolates examined	Rugose conversion frequency^1^ (%)	
				25°C	37°C
*V. cholerae* O1	Clinical	2010	14	0 (0)	1 (7.1)
*V. cholerae* O1	Clinical	2012	27	6 (22.2)	17 (63.0)
*V. cholerae* O1	Clinical	2013	29	4 (13.8)	11 (37.9)
*V. cholerae* O1	Environmental	2012	7	2 (28.6)	5 (71.4)
*V. cholerae* O1	Environmental	2013	17	4 (23.5)	7 (41.2)
*V. cholerae* non-O1/non-O139	Environmental	2012	54	5 (9.3)	28 (51.9)
*V. cholerae* non-O1/non-O139	Environmental	2013	24	2 (8.3)	10 (41.7)
Total			172	23(13.4) *	79(45.9) *

1 Number of strains converted from smooth to rugose phenotype is included. With the exception of one clinical strain, all strains that exhibited a rugose phenotype at 25°C also expressed the rugose phenotype at 37°C (please see Table S1).

*Differences between rates of rugose conversion at 25°C and 37°C are statistically significant

### Effect of temperature on rugose colony morphology

To determine the effect of temperature on rugose colony phenotype and to see if rugose phenotypes of N16961R and HC16R (altered Haitian rugose variant of *V. cholerae*) respond differently to different temperatures, we inoculated a single colony of each strain, grown overnight at 37°C on L-agar, in L-broth and the cultures were incubated overnight at 37°C with a shaking speed of 250 rpm. The cultures were serially diluted in sterile 0.85% saline and an appropriate dilution was spread on two L-agar plates: one plate was incubated at 37°C and the second plate was incubated at 42°C. All the plates were incubated for 72 h, and the rugose cultures grown on L-agar were monitored each day visually and by an Illuminated Stereo Microscope (Leica Zoom 2000, Buffalo, NY) to examine whether the rugose colony phenotype was affected by incubation temperature.

### Genetic manipulations

VpsA (VC_0917, encoding UDP-N-acetylglucosamine 2-epimerase [*wecB*]) [17] is required for the biosynthesis of rugose exopolysaccharide production and to elicit the rugose colony phenotype. To determine if VpsA is essential for the expression of rugose colony phenotype in Haitian altered *V. cholerae* strain, we created a *vpsA* in-frame deletion mutation in the background of HC16R strain (Table 4), using genetic manipulations described recently [18]. An internal in-frame deletion in the *vpsA* [HC16RΔ*vpsA* (Table 4)] was created and the mutation was verified by PCR and DNA sequencing. To revert *in trans* the defect of the *vpsA* mutant, a wild-type *vpsA* gene from N16961S strain was cloned into pWSK29 resulting in a plasmid pAA99; subsequently the plasmid pAA99 was electroporated into the *vpsA* mutant as described previously [19]

**Table 4:** Bacterial strains and plasmids used in this study.

Strain or Plasmid	Description	Reference
***V. cholerae*** ** strains**		
N16961S	A wild-type, smooth, O1 El Tor strain isolated in Bangladesh in 1971	[8]
N16961R	A rugose variant of N16961S strain	[8]
HC16S	A smooth variant of clinical *V. cholerae* strain isolated in 2012 from Haiti	This study
HC16R	A rugose variant of HC16S strain induced in APW #3	This study
AA260 (HC16RΔ*vpsA*)	A *vpsA* gene deletion mutant created in the back ground of HC16R strain.	This study
AA262 (HC16RΔ*vpsA*/pAA99)	The strain AA260 was transformed with pAA99 carrying wild-type *vpsA* gene cloned into pWSK29 (complementing vector)	This study
***E. coli*** ** strains**		
DH5α	*recA* Δ*lac*U169 φ80d *lacZ*ΔM15	Gibco, BRL
S17-1 λ *pir*	*Pro hsdR hsdM* ^+^ Tmp^r^ Str^r^	[29]
**Plasmids**		
pWSK29	Low-copy-number vector, Amp^r^, *ori* pSC101	[30]
pCVD442	Suicide vector, *ori* R6K, Amp^r^, *sacB*	[31]
pAA72	A 540-bp PCR fragment (*SacII-SpeI*) fragment upstream of *vpsA* gene of N16961 cloned into similarly digested pWSK29, Amp^r^	[18]
pAA74	A 360-bp PCR fragment (*SpeI-EcoR1*) downstream of *vpsA* was cloned into similarly digested pAA72, resulting in a plasmid (pAA74). Amp^r^	[18]
pAA77	A 900-bp PCR fragment (*SacI-SalI*) from pAA74 was cloned into similarly digested pCVD442, Amp^r^	[18]
pAA99	A 1.8 kb fragment (*SacII-EcoR1*) of wild-type *vpsA* gene was cloned into similarly digested pWSK29, Amp^r^	[18]

### Biofilm assays

Quantitative assessment of biofilm produced by *V. cholerae* strains was measured as described previously [20]. Briefly, twenty-four well polystyrene plastic plates (Corning Incorporated, Corning, NY) were used as the surface for bacterial attachment. Wild-type *V. cholerae* strain HC16S and its isogenic variant/mutants, including HC16R, HC16RΔ*vpsA* and HC16RΔ*vpsA*/pAA99 (Table 4) was included in the biofilm assay; N16961S and N16961R were used as control strains. The microorganisms were grown in L-broth and incubated overnight statically at room temperature. Following overnight incubation the cultures were discarded, and the tubes were then rinsed vigorously with distilled water to remove non-adherent cells, filled with 600 µl of a 0.1% crystal violet solution (Sigma, St. Louis, MO), allowed to incubate for 30 min at room temperature, and the tubes were again rinsed vigorously with water. Quantitative biofilm formation was determined by measuring the optical density at 570 nm (OD 570) of a solution produced by extracting cell-associated dye with 600 µl of dimethyl sulfoxide (DMSO) (Sigma, St. Louis, MO).

### Confocal microscopy

To determine three dimensional and architectural structure of biofilm formation by *V. cholerae* strains, laser scanning confocal microscopic (LSCM) analysis was performed [18]. Briefly, the biofilms of *V. cholerae* strains were allowed to develop on 75 X 25 mm glass slides (Corning Inc., Corning, NY), and dipped in L-broth incubated overnight statically at 37°C. After appropriately staining and follow-up processing of the slides, the biofilm thickness was measured as an average of 5 non-overlapping fields per slide with a 20X HCX PL APO lambda blue magnifying objective. Images were digitally reconstructed with z-projections of x-y sections using Leica Application Suite Advanced Fluorescence (Leica Microsystem, Buffalo Grove, IL) and DAIME softwares [21]. The volumes of biofilms were calculated as follows: the x-y areas of each z-section were measured using Image J (National Institute of Mental Health, Bethesda, Maryland, USA) and were multiplied by the value of the z-step to obtain the volume of the biofilm at each section. Total biofilm volumes were calculated as a sum of the separate volumes of the z-sections as described previously[22]. At least two biological replicates were used in the imaging processes.

### Stress resistance assay

*V. cholerae's* resistances to stress, including both oxidative and osmotic stress and chlorine resistance was assessed as described earlier [8], [9]. *V. cholerae* strain HC16S and its isogenic variant/mutants, including HC16R and 16RΔ*vpsA*, were included in the stress resistance assay. In addition, *V. cholerae* strains N16961S and N16961R were used as negative and positive controls, respectively. A single colony of overnight grown *V. cholerae* strain was inoculated into 3 ml L-broth and the culture was incubated overnight at 37°C with a shaking speed of 250 rpm. The culture was spun down and the pellet was washed 2X with phosphate buffered saline (PBS, pH 7.4) and subsequently the culture was reconstituted into 3 ml PBS. A ten-fold dilution of the culture was made into 3 ml PBS to obtain ca. 10^8^ cfu/ml and the culture was subjected to stress resistance assays as follows: For oxidative stress, 20 mM H2O2 (final concentration) (hydrogen peroxide 3%, Ricca Chemical, Arlington, TX) was added to each culture and the resistance of each culture to H2O2 was recorded for every 5 minutes for 20 min. The culturable bacteria survived the stress was determined using standard plate count at each experimental time point. Similarly, for osmotic and chlorine stresses, *V. cholerae's* culture in PBS was exposed to 2.5 M NaCl (at final concentration) (Avantor Performance Materials, Center Valley, PA) and 3 mg free chlorine per liter [3 ppm] (sodium hypochlorite, Sigma, St Louis, MO), respectively. Resistance of each *V. cholerae* strain to osmotic and chlorine stresses were determined by measuring the culturable bacteria present (i) for every 15 min for one hour [9], and (ii) for every 5 min for 20 min [8], respectively. Percent survival of the bacteria was calculated by dividing the number of bacterial colonies counted at a given time by the number of microorganisms added to the culture before supplementing the culture with stress ingredient, and then multiplying the result by 100.

### Phylogenetic analysis

To assess the phylogenetic relationship of rugose and non-rugose producing strains, we assessed whole-genome sequencesof 65 *V. cholerae* O1 isolates, including 42 clinical isolates and 21environmental isolates; results of this sequence analysis for factors other than rugose production are reported elsewhere (Azarian *et al*, 2014-submitted). Seventeen clinical isolates and 8 environmental isolates were rugose producing, and 25 clinical isolates and 13 environmental isolates were rugose negative. Three whole-genome sequences of historical Nepalese *V. cholerae* O1 isolates were used to root the phylogenetic tree. Whole-genome sequencing and phylogenetic analysis were performed as previously described (Azarian et al. 2014-submitted). Briefly, genome-wide SNPs were extracted through our bioinformatics protocol and high quality SNPs were identified with a depth of coverage above 10 and a minimum alternate allele fraction of 0.75. SNP sites with ambiguities were removed to produce a final filtered SNP alignment. Genome-wide SNPs were phylogenetically analyzed using MEGA5 [23]. Maximum likelihood phylogenies were constructed using a Kimura 2-parameter nucleotide substitution model and 1000 bootstrap replicates. Phylogenies were rooted using three closely related Nepalese strains[24]. Between and within group genetic distances were also calculated for rugose and non-rugose producing strains. The phylogenetic relationship between the N16961 reference strain (RefSeq accession numbers NC_002505.1 and NC_002506.1) and O395 reference strain (RefSeq accession numbers NC_009456.1 and NC_009457.1) were compared to the Haitian cluster. Last, phylogenetic compartmentalization, i.e. whether rugose and non-rugose producing strains cluster on distinct clades of the phylogeny, was tested using the tree correlation coefficient test and Slatkin Maddison test implemented in HyPhy [25].

### Statistical analysis

Simple logistic regression was used to determine if the proportion of smooth *V. cholerae* isolates that were able to switch to rugose phenotypes were significantly different between years of isolation and origin of isolation (clinical or environmental). For each isolated strain, McNemar's test was used to determine if the marginal differences between the rate of conversion from smooth to rugose phenotypes were significantly different between incubation temperatures (25 or 37°C). Finally, one-way ANOVA was performed to determine if the differences in traits associated with biofilm density and resistance to oxidative stress were statistically significant. A p-value of <0.05 was considered statistically significant. All statistical tests were performed using STATA v12 (StataCorp, College Station Texas, USA).

## Results

### Rugose production by Haitian *V. cholerae* strains

During our survey for the detection of environmental *V. cholerae* isolates in Haiti, we spread water samples directly onto L-agar (in addition to conventional approach to detect *V. cholerae* involving APW enrichment followed by the streaking of the culture onto selective TCBS agar) in order to determine total heterotrophic bacterial counts and the plates were incubated overnight at 37°C and then left at room temperature for at least additional 3 days. We observed that <1% of the samples yielded *V. cholerae* rugose phenotype on L-agar after at least 48 h of incubation. This observation led us to undertake these current investigations. A total of 172 *V. cholerae* O1 and non-O1/non-O139 strains from clinical and environmental sources in Haiti were examined to evaluate their ability to shift to a rugose colony phenotype (Table S1 and Table 1). Of 172 strains, 80 (46.5%) switched from smooth to rugose phenotype on L-agar following their growth in APW incubated either at 25°C or 37°C (Table 1). Out of 80 rugose positive strains, 68 (85%) switched to a rugose phenotype only after their growth on L-agar for at least 48 h; 12 (15%) strains switched to rugose phenotype at 24 h of incubation on L-agar (Table S1) as is generally seen with typical *V. cholerae* O1 El Tor strains, including N16961S. Consistent with a previous report, *V. cholerae* O1 Classical biotype strain (O395) was unable to shift to a rugose phenotype [8].

Out of 70 clinical O1 strains collected in 2010, 2012 and 2013, 30 (42.9%) strains switched to a rugose colony phenotype (Table 1). When results are broken out by year, only 1 (7.1%) of 14 strains isolated in 2010, 18 (66.7%) of 27 strains isolated in 2012, and 11 (37.9%) of 29 strains isolated in 2013 were able to switch to a rugose phenotype (Table 2). Compared to clinical isolates of 2010, clinical isolates obtained in 2012 and 2013 were significantly more likely (p = 0.014) to convert from smooth to rugose phenotypes. Environmental isolates were only available for 2012 and 2013; for these years, differences among clinical, environmental O1, and environmental non-O1/non-O139 strains were not statistically significant. Differences in percent of total colonies of each strain that assumed a rugose phenotype did not differ significantly among strains based on source (clinical, environmental O1, environmental non-O1/non-O139) or year of isolation.

### Effect of temperature on rugose colony phenotype expression

To determine the effect of incubation temperatures on the switching of smooth colony phenotype to rugose phenotype, in otherwise identical growth conditions, all 172 *V. cholerae* isolates were grown in APW broth at 25°C and 37°C for 72 h prior to plating them on L-agar (Table S1). Out of 172 strains, only 23 (13.4%) produced the rugose colony phenotype at 25°C while 79 (45.9%) produced the rugose phenotype at 37°C (Table 3). With one exception, all strains that converted to a rugose phenotype at 25°C also converted to rugose at 37°C; in contrast, 56 strains produced a rugose phenotype only at 37°C, but not 25°C (p<0.0001) (Table S1). In a subgroup analysis, the increase in rugose conversion at 37°C remained significant for all clinical, environmental O1, and environmental non-O1/non-O139 strains. The difference in smooth to rugose conversion was also observed for the typical smooth *V. cholerae* N16961 strain which switched from smooth to rugose colony phenotype to 30% and 80% at 25°C and at 37°C, respectively (Table S1).

**Figure 1:**
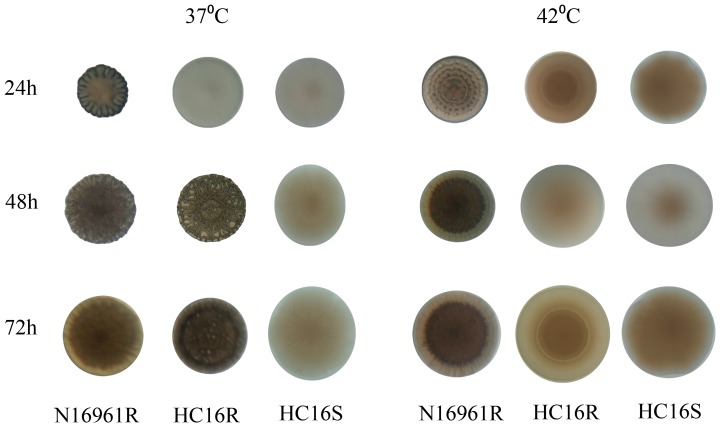
Effect of temperatures on the colony phenotype of rugose variant of *V. cholerae* strains grown on L-agar.

To further determine the effect of temperatures on rugose colony phenotype, both a rugose variant of *V. cholerae* N16961R and a Haitian rugose strain (HC16R) were spread on L-agar plates and the plates were incubated at 37 and 42°C for 72 h. As shown in Figure 1, the HC16R colony phenotype, in contrast to N16961R colony phenotype, switched to a smooth colony phenotype and remained smooth for at least 72 h at 42°C. Furthermore, when grown at 37°C, the HC16R rugose colony phenotype compared to N16961R colony phenotype reverted to a smooth phenotype and remained smooth for 24 h on L-agar after which the smooth colony switched back to a rugose colony phenotype (Fig. 1).

### Biofilm formation

**Figure 2:**
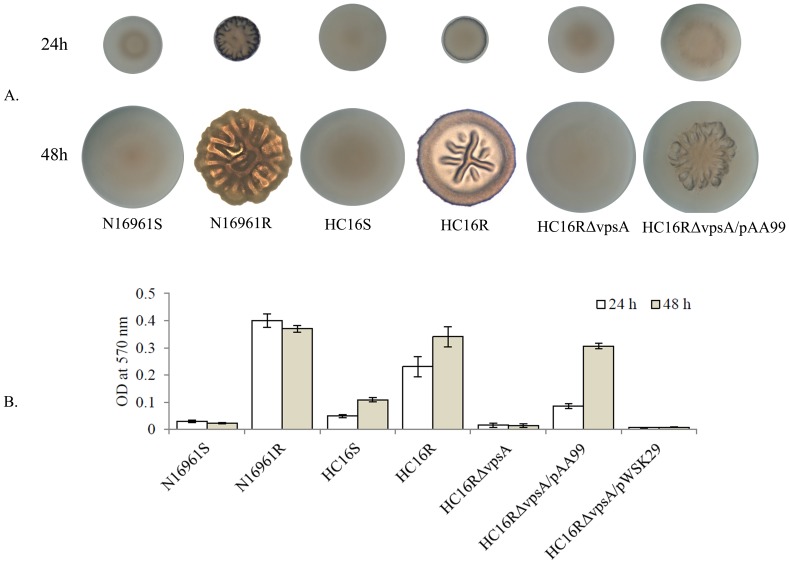
Colony morphology and associated biofilms (measured quantitatively) produced by *V. cholerae* wild-type and mutant strains. (A) Colony morphology at 24 h and 48 h: each *V. cholerae* strain was subcultured on L-agar for isolated colonies, and the culture plate was incubated overnight at 37°C for 48 h. The images of bacterial colony were taken at 24 and 48 h of incubations; (B) Quantitative measurement of biofilm produced by each *V. cholerae* strain in nutrient-rich L-broth. All the values are expressed as means ± standard deviation (SD) from at least triplicate experiments.

In *V. cholerae*, rugose colony phenotype, exopolysaccharide production and biofilm formation are a mutually inclusive process [26]. Previous study demonstrated that VpsA encoded by *vpsA* gene is required for rugose colony phenotype and biofilm formation [17]. To determine whether *vpsA* is also required for rugose colony phenotype and biofilm formation in a Haitian altered *V. cholerae* strain, we created an in-frame null mutation in a *V. cholerae* strain (HC16R). Since both smooth and rugose variants of *V. cholerae* generally stably maintained their respective colony phenotype on L-agar, we examined colony phenotypes of *V. cholerae* cells grown on L-agar for 24 and 48 h. As expected, smooth variants of N16961S and HC16S exhibited smooth colony phenotypes on L-agar even after 48 h of growth. Moreover, N16961R produced a pronounced rugose colony phenotype as early as 24 h of growth; interestingly, HC16R exhibited a smooth-like colony phenotype at 24 h, which progressed to a more fully developed rugose phenotype after 48 h (Fig. 2A). In contrast, a null mutation in the *vpsA* gene in HC16R inhibited expression of the rugose colony phenotype even after 48 h of growth. The expression of the wild-type *vpsA* gene *in trans* on a plasmid (pAA99) (Table 4) resulted in reversion of the mutant to the wild-type rugose phenotype. These observations confirm that *vpsA* is required for the expression of rugose colony phenotype in HC16R.

We also measured the quantitative biofilm production by *V. cholerae* strains using methods as described previously [20]. *V. cholerae* N16961R rugose strains produced significantly more (10-fold) biofilm by 48 h compared to its isogenic smooth (N16961S) counterpart (p<0.001); HC16R exhibited more than a 3-fold increase in biofilm production by 48 h compared to its smooth counterpart HC16S (p<0.001). As expected the HC16R *vpsA* mutant was unable to form biofilm; however, the reverted mutant (complemented strain) produced almost equal amounts of biofilm as seen with HC16R by 48 h of growth (Fig. 2B).

### Confocal microscopy

**Figure 3:**
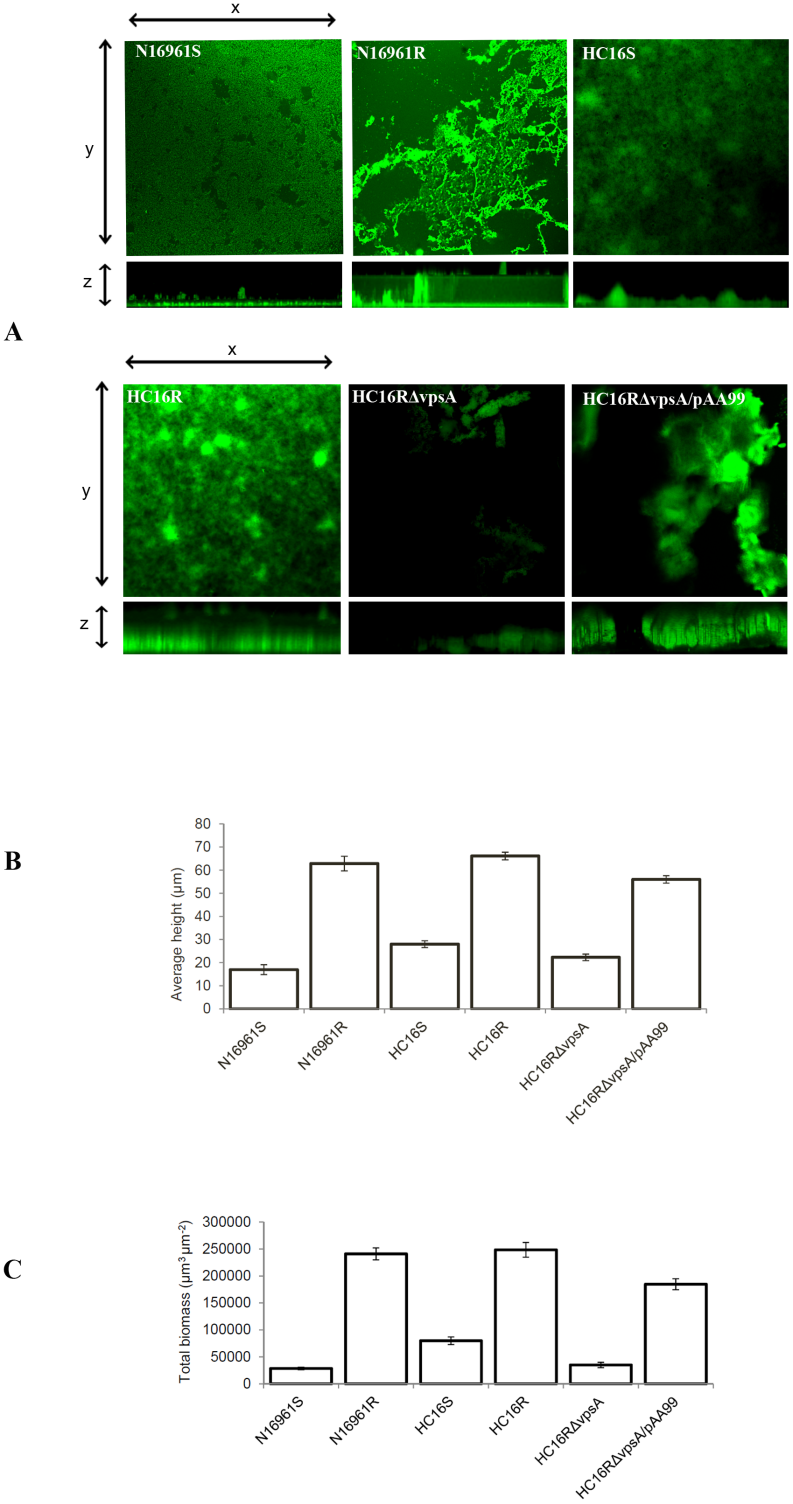
Topography and architecture of *V. cholerae* biofilms. Each strain was grown in a 4-well cell culture plate containing 500 µl L-broth. A glass cover slip was dipped into each culture well and incubated overnight statically at room temperature. The glass cover slips were stained with SYTO 9 and the images were obtained using a laser scanning confocal microscopy with an excitation and emission wavelengths of 484- and 500 nm, respectively, as described previously [18]. (A) Images of x-y sections (top portions of panels) and x-z projections of the same biofilms (bottom portions of panels) were analyzed with DAIME software; magnification, ×200. (B) Average biofilm heights (µm) for each strain measured across five random x-z sections. (C) Total biomass of biofilm (µm^3^µm^−2^) for each strain calculated by x-y and x-z projections.

To compare the three dimensional architectural structures of biofilm produced in L-broth for 48 h by HC16S and N16961S and their isogenic rugose variants/mutant, we examined biofilm using SCLM. While N16961R formed a generally uniform biofilm matrix, HC16R formed biofilms with galaxy-like structures with bulge and halos dispersed across the biofilm matrix (Fig. 3A). The rugose variants of HC16R and N16961R produced highly-developed structured and matured biofilm exhibiting multicellular matrix with 66 and 63 µm high pillars and columns filled with fluids, respectively. In contrast, the smooth variants of HC16S and N16961S displayed a less-developed (mostly monolayers) biofilm with 28 and 17 µm pillars, respectively. While HC16RΔ*vpsA* produced as much biofilm as seen with HC16S (22 vs 28 µm), HC16RΔ*vpsA*/pAA99 restored biofilm in height and volume to the level seen with wild-type HC16R biofilm with 56 µm pillars filled with fluids (Figs. 3B and 3C).

### Stress resistance

**Figure 4:**
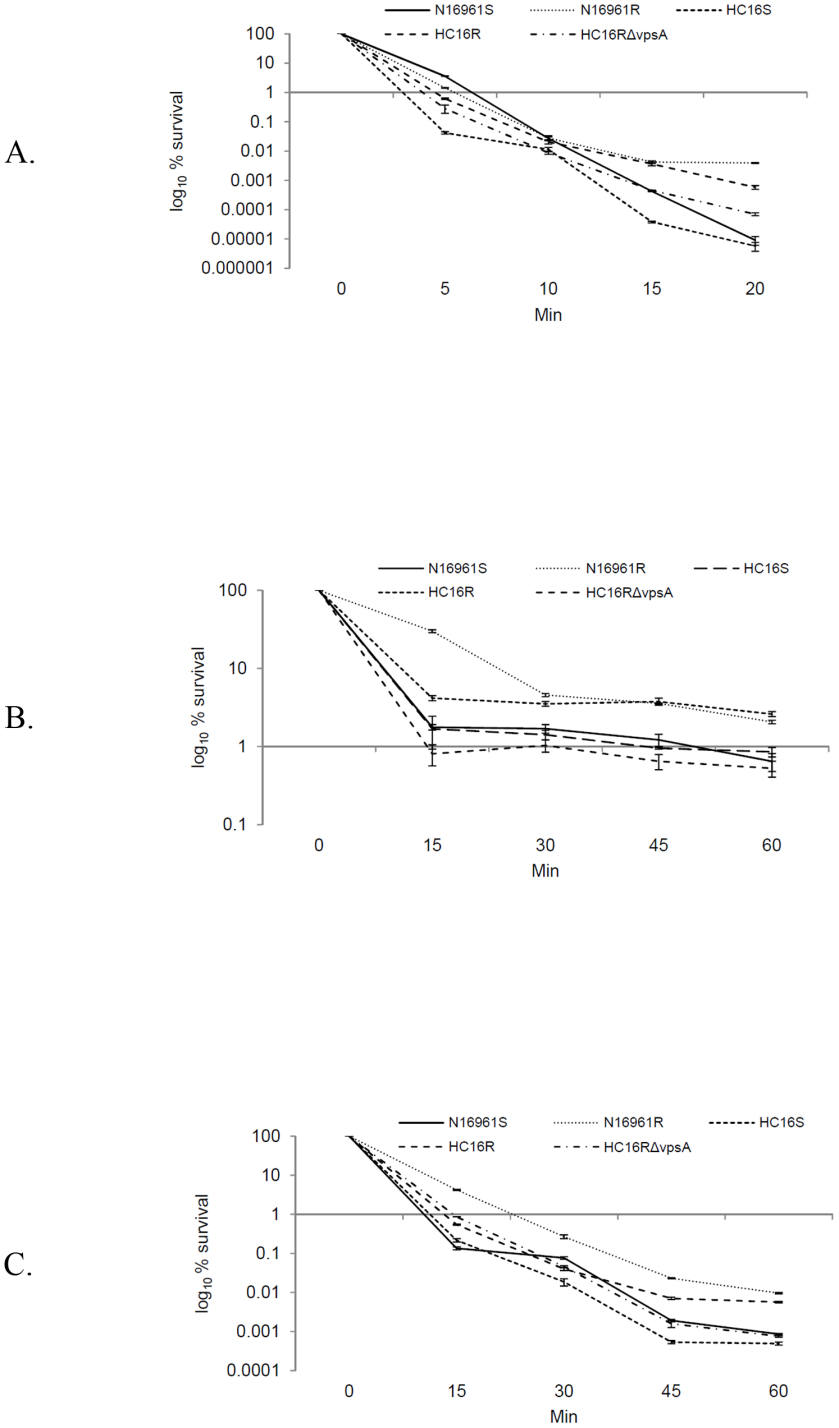
Susceptibility of *V. cholerae* strains to diverse environmental stresses: (A) survival of the strains in the presence of 20 mM H_2_O_2_; (B) 2.5 M NaCl; and (C) 3 mg free chlorine/L. The strains were grown (ca. 10^8^ cfu/ml) in L-broth, washed and reconstituted with phosphate buffered saline (PBS). Stress assays were conducted in PBS supplemented with stress ingredients. The cultures were examined at different time intervals for the presence of bacteria as determined by standard plate count. Error bars indicate means ± standard deviation (SD) from triplicate experiments.

We investigated whether the Haitian *V. cholerae* rugose variant was resistant to the stresses as seen and previously reported for *V. cholerae* [9]. As shown in Figure 4, Haiti rugose variant (HC16R) as well as the N16961R strain exhibited significantly increased resistance (p value <0.001) to H_2_O_2_ (Fig. 4A), NaCl (Fig 4B), and NaOCl (Fig 4C) compared to their smooth counterparts N16961S and HC16S, respectively. As expected, HC16RΔ*vpsA* was sensitive to distinct stresses and exhibited responses similar to and not significantly different (p value >0.5) from its smooth counterpart HC16S and N16961S. Our observations further demonstrate that VPS confers resistance to diverse types of environmental stress.

### Phylogenetic analysis

**Figure 5:**
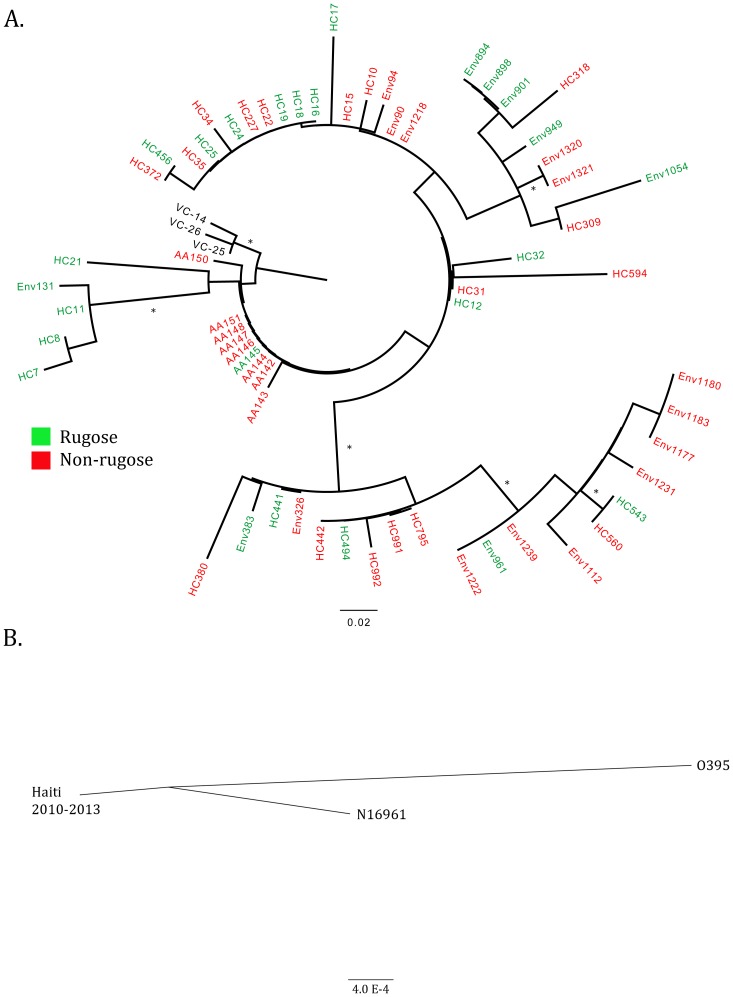
Phylogenies of rugose and non-rugose producing O1 *V. cholerae* strains isolated in Haiti. (A) Maximum likelihood phylogenetic tree of rugose (green) and non-rugose (red) producing clinical and environmental strains. Phylogeny was constructed with MEGA5 using the Kimura 2-parameter nucleotide substitution model with 1,000 bootstrap replicates. Clades with bootstrap support greater than 70 are indicated with an asterisk. Clinical strains are prefixed with AA or HC and environmental strains are prefixed with Env., (B) Neighbor-joining phylogeny of Haitian strains displaying the global relationship to the N16961 and O396 reference strains.

Figure 5A depicts the phylogenetic relationship between the rugose and non-rugose-producing clinical and environmental strains. The relationship of the Haitian *V. cholerae* O1 epidemic clone and the N16961 and O395 reference strains are depicted in Figure 5B. The between group mean genetic distance was 0.11, while the mean within group genetic distance was 0.12 among the rugose producing strains and 0.10 among the non-rugose producing strains. Descriptively, some clades (e.g. including isolates HC7, HC8, HC11, HC21, env131) appeared to cluster based on the ability to shift to a rugose phenotype. However, when statistically assessing the compartmentalization of the phylogeny, rugose and non-rugose producing strains did not cluster significantly when tested using the tree correlation coefficient (p value >0.05) or the Slatkin Maddison test (p value >0.05).

## Discussion

After 100 years of absence, toxigenic *V. cholerae* O1 appears to have been accidentally introduced in Haiti in October, 2010 and the epidemic, though showing signs of reduced intensity, is still continuing. We recently reported that toxigenic *V. cholerae* has established possible aquatic reservoirs in the Gressier/Leogane regions in Haiti [15].

It is still early to draw any conclusions regarding long-term persistence of toxigenic *V. cholerae* in the environment in Haiti. However, in evaluating this risk it is important to understand the characteristics of the circulating strains, particularly with regard to their ability to shift to a phenotype capable of surviving a range of environmental stressors.

In this report we provide evidence that (i) 46.5% of *V. cholerae* strains examined were able to shift to rugose colony phenotype, (ii) in contrast to typical N16961 smooth strain (N16961S, El Tor biotype) that switched to rugose phenotype by 24 h, the majority of Haitian smooth strains switched to a rugose colony phenotype only after their growth on L-agar for at least 48 h, (iii) incubation temperatures influenced the expression of rugose colony phenotype of Haitian *V. cholerae* strain (HC16R), in contrast to typical N16961R strain, and (iv) unlike our previous report [8] where we demonstrated that rugose production was significantly higher among clinical isolates compared to that of among environmental isolates, Haitian *V. cholerae* strains exhibited similar frequency in rugose colony phenotype expression between clinical and environmental isolates. Taken together our observations suggest that N16961S and Haitian *V. cholerae* smooth strains require distinct growth conditions for S–R conversion, and that incubation time and temperatures play a critical role in the expression of rugose production by Haitian strains with 37°C favored while 25°C and 42°C (in limited studies) inhibited that phenotype. Interestingly and in contrast to Haitian *V. cholerae* strains, *Vibrio vulnificus* [27] and *Salmonella enterica* [28] favored the expression of rugose colony phenotypes in 30°C and 25°C, respectively. It is possible that Haitian *V. cholerae* may adopt alternative survival strategies, including “persister” phenotype [6] and viable but non-culturable (VBNC) state [5] when it persists at lower temperature.

We and others have previously reported that *V. cholerae* rugose variants compared to their smooth counterparts can resist chlorine, oxidative, and osmotic stresses [7], [8], [9], and that, the stress resistance is attributed to the ability of rugose variants to produce exopolysaccharide. Consistent with those previous reports, we demonstrated that HC16R was resistant to both oxidative, osmotic and chlorine stresses (Figs. 4A, B and C). Using SCLM we demonstrated that Haitian *V. cholerae* O1 strain HC16R has distinct biofilm architectural structure than that of N16961R strain (Fig. 3). However, without compositional analysis of biofilms, we cannot infer whether HC16R and N16961R biofilms are different in regard to their biofilm matrix compositions. Even if it turns out that HC16R biofilm is different in matrix composition relative to N16961R biofilm, the roles of HC16R biofilm is similar to N16961R in relation to conferring resistance to diverse stresses (Fig. 4).

Of particular interest, we observed that while only 7.1% of clinical strains isolated in 2010 switched to rugose phenotype, 66.7% and 37.9% clinical isolates collected in 2012 and 2013, respectively, shifted to that phenotype (Table 2). Although we have examined a limited number of strains (n = 14) from 2010, our observations raise the possibility that smooth clinical O1 strains of *V. cholerae* are evolving to the rugose variant seemingly to better adapt to Haitian aquatic reservoirs. Indeed, endogenous environmental *V. cholerae* O1 and non-O1/non-O139 strains isolated in 2012 and 2013 in Haiti exhibited a similar pattern of rugose production (41.7–51.9%)[Table 2] that was parallel to clinical O1 strains isolated in 2012 and 2013.

As an initial step in exploring evolutionary changes in these strains, we assessed the phylogeny of the clinical and environmental rugose and non-rugose producing strains to investigate a genotypic relationship for the observed phenotype (Fig. 5). We found that while there were distinct clusters of rugose producing strains on the phylogeny; these clusters were not statistically significant. This suggests a panmictic population structure in regard to the rugose phenotype. Interestingly, there appear to be some clades in which environmentally sampled rugose-producing strains are located basal to clinically sampled isolates. There may indeed be other underlying mechanisms for rugose production, including transcriptional, translational and post-transcriptional/translational genetic mechanisms, and the topology of the phylogeny may represent other geographical or temporal characteristics that will need to be investigated further. Also, the SNPs assessed represent only portion of the genetic variation between strains. For example, insertions and deletions (INDELs) as well as multi-nucleotide polymorphisms as well as sites not conserved across all isolates are excluded from the phylogenetic analysis.

In summary, we demonstrated that the altered toxigenic and clonal *V. cholerae* O1 El Tor strain introduced in Haiti in 2010 clearly has the ability to shift to a rugose phenotype, which protects against environmental stress; there is also a suggestion that the ability of strains to shift to this phenotype has increased across time. We propose that rugose variant of *V. cholerae* has adapted to aquatic reservoirs in Haiti and that this phenotype can significantly contribute to the endemicity of cholera in that country, with significant public health implications in regard to establishment of cholera endemicity in Haiti.

## Supporting Information

Table S1 **Rugose conversion rate of** ***V. cholerae*** **strains in Haiti.**
(DOCX)
