# Supplementary material for: High-Frequency Rugose Exopolysaccharide Production by Vibrio cholerae Strains Isolated in Haiti
Source: PLoS One. 2014 Nov 12;9(11):e112853. doi: 10.1371/journal.pone.0112853 (PMC4229229; doi:10.1371/journal.pone.0112853)
Supplement: Table S1 — Rugose conversion rate of V. cholerae strains in Haiti. (DOCX) [file pone.0112853.s001.docx]

Table S1. Rugose conversion rate of *V. cholerae* strains in Haiti

| Sl. No. | Strain | Origin | Serogroup | Collect. year | % Rugose conver.at 25ºC^a^ | % Rugose conver. |
| --- | --- | --- | --- | --- | --- | --- |
|  |  |  |  |  |  | at 37ºC^b^ |
| 1 | AA142 | Clinical | *V. cholerae* O1 | Oct,2010 | 0 | 0 |
| 2 | AA143 | Clinical | *V. cholerae* O1 | Oct,2010 | 0 | 0 |
| 3 | AA144 | Clinical | *V. cholerae* O1 | Oct,2010 | 0 | 0 |
| 4 | AA145 | Clinical | *V. cholerae* O1 | Oct,2010 | 0 | 26.5 |
| 5 | AA146 | Clinical | *V. cholerae* O1 | Oct,2010 | 0 | 0 |
| 6 | AA147 | Clinical | *V. cholerae* O1 | Oct,2010 | 0 | 0 |
| 7 | AA148 | Clinical | *V. cholerae* O1 | Oct,2010 | 0 | 0 |
| 8 | AA150 | Clinical | *V. cholerae* O1 | Oct,2010 | 0 | 0 |
| 9 | AA151 | Clinical | *V. cholerae* O1 | Oct,2010 | 0 | 0 |
| 10 | AA152 | Clinical | *V. cholerae* O1 | Oct,2010 | 0 | 0 |
| 11 | AA153 | Clinical | *V. cholerae* O1 | Oct,2010 | 0 | 0 |
| 12 | AA154 | Clinical | *V. cholerae* O1 | Oct,2010 | 0 | 0 |
| 13 | AA155 | Clinical | *V. cholerae* O1 | Oct,2010 | 0 | 0 |
| 14 | AA156 | Clinical | *V. cholerae* O1 | Oct,2010 | 0 | 0 |
| 15 | HC5 | Clinical | *V. cholerae* O1 | Apr,2012 | 17.7 | 0 |
| 16 | HC7 | Clinical | *V. cholerae* O1 | Apr,2012 | 0 | 2.3 |
| 17 | HC8 | Clinical | *V. cholerae* O1 | Apr,2012 | 9.5 | 13.6 |
| 18 | HC10 | Clinical | *V. cholerae* O1 | Apr,2012 | 0 | 0 |
| 19* | HC11 | Clinical | *V. cholerae* O1 | May,2012 | 0 | 12.0 |
| 20 | HC12 | Clinical | *V. cholerae* O1 | May,2012 | 0 | 24.0 |
| 21 | HC14 | Clinical | *V. cholerae* O1 | May,2012 | 14.7 | 4.6 |
| 22 | HC15 | Clinical | *V. cholerae* O1 | May,2012 | 0 | 2.5 |
| 23 | HC16 | Clinical | *V. cholerae* O1 | May,2012 | 17.3 | 85.0 |
| 24 | HC17 | Clinical | *V. cholerae* O1 | May,2012 | 13.2 | 1.9 |
| 25 | HC18 | Clinical | *V. cholerae* O1 | May,2012 | 0 | 23.5 |
| 26 | HC19 | Clinical | *V. cholerae* O1 | May,2012 | 0 | 0 |
| 27 | HC21 | Clinical | *V. cholerae* O1 | May,2012 | 0 | 57.3 |
| 28 | HC22 | Clinical | *V. cholerae* O1 | May,2012 | 0 | 13.9 |
| 29 | HC23 | Clinical | *V. cholerae* O1 | May,2012 | 0 | 12.5 |
| 30* | HC24 | Clinical | *V. cholerae* O1 | May,2012 | 6.0 | 33.1 |
| 31* | HC25 | Clinical | *V. cholerae* O1 | May,2012 | 0 | 58.2 |
| 32 | HC31 | Clinical | *V. cholerae* O1 | June,2012 | 0 | 0 |
| 33* | HC32 | Clinical | *V. cholerae* O1 | June,2012 | 0 | 47.6 |
| 34 | HC34 | Clinical | *V. cholerae* O1 | June,2012 | 0 | 0 |
| 35 | HC35 | Clinical | *V. cholerae* O1 | June,2012 | 0 | 0 |
| 36 | HC120 | Clinical | *V. cholerae* O1 | Sept,2012 | 0 | 0 |
| 37 | HC121 | Clinical | *V. cholerae* O1 | Sept,2012 | 0 | 0 |
| 38 | HC128 | Clinical | *V. cholerae* O1 | Oct,2012 | 0 | 8.6 |
| 39 | HC129 | Clinical | *V. cholerae* O1 | Oct,2012 | 0 | 0 |
| 40 | HC141 | Clinical | *V. cholerae* O1 | Oct,2012 | 0 | 0 |
| 41 | HC157 | Clinical | *V. cholerae* O1 | Nov,2012 | 0 | 3.3 |
| 42 | HC227 | Clinical | *V. cholerae* O1 | June,2013 | 0 | 0 |
| 43 | HC309 | Clinical | *V. cholerae* O1 | June,2013 | 0 | 0 |
| 44 | HC318 | Clinical | *V. cholerae* O1 | June,2013 | 0 | 0 |
| 45 | HC330 | Clinical | *V. cholerae* O1 | July,2013 | 0 | 5.3 |
| 46 | HC331 | Clinical | *V. cholerae* O1 | July,2013 | 0 | 3.2 |
| 47 | HC372 | Clinical | *V. cholerae* O1 | July,2013 | 0 | 0 |
| 48 | HC380 | Clinical | *V. cholerae* O1 | July,2013 | 0 | 0 |
| 49 | HC441 | Clinical | *V. cholerae* O1 | Aug,2013 | 0 | 39.0 |
| 50 | HC442 | Clinical | *V. cholerae* O1 | Aug,2013 | 0 | 0 |
| 51 | HC456 | Clinical | *V. cholerae* O1 | Aug,2013 | 0 | 50.0 |
| 52 | HC494 | Clinical | *V. cholerae* O1 | Aug,2013 | 0 | 6.4 |
| 53 | HC543 | Clinical | *V. cholerae* O1 | Sept,2013 | 0.4 | 7.2 |
| 54 | HC547 | Clinical | *V. cholerae* O1 | Sept,2013 | 1.1 | 20.0 |
| 55 | HC560 | Clinical | *V. cholerae* O1 | Sept,2013 | 0 | 0 |
| 56 | HC594 | Clinical | *V. cholerae* O1 | Sept,2013 | 0 | 0 |
| 57 | HC635 | Clinical | *V. cholerae* O1 | Oct,2013 | 0 | 8.0 |
| 58 | HC636 | Clinical | *V. cholerae* O1 | Oct,2013 | 0 | 0 |
| 59 | HC637 | Clinical | *V. cholerae* O1 | Oct,2013 | 0.1 | 6.1 |
| 60 | HC646 | Clinical | *V. cholerae* O1 | Oct,2013 | 0 | 0 |
| 61 | HC784 | Clinical | *V. cholerae* O1 | Nov,2013 | 0 | 0 |
| 62 | HC785 | Clinical | *V. cholerae* O1 | Nov,2013 | 0 | 0 |
| 63 | HC795 | Clinical | *V. cholerae* O1 | Nov,2013 | 0 | 0 |
| 64 | HC909 | Clinical | *V. cholerae* O1 | Nov,2013 | 0 | 0 |
| 65 | HC945 | Clinical | *V. cholerae* O1 | Dec,2013 | 0 | 0 |
| 66 | HC946 | Clinical | *V. cholerae* O1 | Dec,2013 | 0 | 15.0 |
| 67 | HC956 | Clinical | *V. cholerae* O1 | Dec,2013 | 0.3 | 6.8 |
| 68 | HC991 | Clinical | *V. cholerae* O1 | Dec,2013 | 0 | 0 |
| 69 | HC992 | Clinical | *V. cholerae* O1 | Dec,2013 | 0 | 0 |
| 70 | HC994 | Clinical | *V. cholerae* O1 | Dec,2013 | 0 | 0 |
| 71 | env9 | Environmental | *V. cholerae* O1 | Apr,2012 | 0 | 0.3 |
| 72 | env90 | Environmental | *V. cholerae* O1 | May,2012 | 0 | 0.8 |
| 73 | env94 | Environmental | *V. cholerae* O1 | May,2012 | 0 | 0 |
| 74 | env131 | Environmental | *V. cholerae* O1 | June,2012 | 0 | 59.5 |
| 75 | env326 | Environmental | *V. cholerae* O1 | Aug,2012 | 0 | 0 |
| 76 | env383 | Environmental | *V. cholerae* O1 | Sept,2012 | 46.7 | 35.1 |
| 77 | env390 | Environmental | *V. cholerae* O1 | Sept,2012 | 51.2 | 62.5 |
| 78 | env122 | Environmental | *V. cholerae* O1 | Feb,2013 | 0 | 0 |
| 79 | env894 | Environmental | *V. cholerae* O1 | June,2013 | 60.0 | 57.1 |
| 80 | env898 | Environmental | *V. cholerae* O1 | June,2013 | 50.0 | 80.0 |
| 81 | env901 | Environmental | *V. cholerae* O1 | June,2013 | 0 | 58.8 |
| 82 | env949 | Environmental | *V. cholerae* O1 | July,2013 | 70.2 | 78.8 |
| 83 | env961 | Environmental | *V. cholerae* O1 | July,2013 | 68.5 | 20.8 |
| 84 | env1054 | Environmental | *V. cholerae* O1 | Aug,2013 | 0 | 1.0 |
| 85 | env1112 | Environmental | *V. cholerae* O1 | Sept,2013 | 0 | 0 |
| 86 | env1177 | Environmental | *V. cholerae* O1 | Sept,2013 | 0 | 0 |
| 87 | env1180 | Environmental | *V. cholerae* O1 | Sept,2013 | 0 | 0.6 |
| 88 | env1183 | Environmental | *V. cholerae* O1 | Sept,2013 | 0 | 0 |
| 89 | env1218 | Environmental | *V. cholerae* O1 | Oct,2013 | 0 | 0 |
| 90 | env1222 | Environmental | *V. cholerae* O1 | Oct,2013 | 0 | 0 |
| 91 | env1231 | Environmental | *V. cholerae* O1 | Oct,2013 | 0 | 0 |
| 92 | env1239 | Environmental | *V. cholerae* O1 | Oct,2013 | 0 | 0 |
| 93 | env1320 | Environmental | *V. cholerae* O1 | Nov,2013 | 0 | 0 |
| 94 | env1321 | Environmental | *V. cholerae* O1 | Nov,2013 | 0 | 0 |
| 95* | env2 | Environmental | *V. cholerae* Non-O1/ Non-O139 | Apr,2012 | 0 | 80.0 |
| 96 | env15 | Environmental | *V. cholerae* Non-O1/ Non-O139 | Apr,2012 | 0 | 0 |
| 97 | env25 | Environmental | *V. cholerae* Non-O1/ Non-O139 | Apr,2012 | 0 | 0 |
| 98 | env26 | Environmental | *V. cholerae* Non-O1/ Non-O139 | Apr,2012 | 0 | 28.3 |
| 99 | env31 | Environmental | *V. cholerae* Non-O1/ Non-O139 | Apr,2012 | 0 | 50.6 |
| 100 | env32 | Environmental | *V. cholerae* Non-O1/ Non-O139 | Apr,2012 | 0 | 18.2 |
| 101 | env70 | Environmental | *V. cholerae* Non-O1/ Non-O139 | May,2012 | 0 | 0 |
| 102 | env82 | Environmental | *V. cholerae* Non-O1/ Non-O139 | May,2012 | 0 | 0 |
| 103 | env92 | Environmental | *V. cholerae* Non-O1/ Non-O139 | May,2012 | 0 | 4.5 |
| 104 | env103 | Environmental | *V. cholerae* Non-O1/ Non-O139 | May,2012 | 0 | 0 |
| 105 | env104 | Environmental | *V. cholerae* Non-O1/ Non-O139 | May,2012 | 0 | 1.0 |
| 106 | env113 | Environmental | *V. cholerae* Non-O1/ Non-O139 | May,2012 | 0 | 0 |
| 107 | env115A | Environmental | *V. cholerae* Non-O1/ Non-O139 | May,2012 | 0 | 0 |
| 108 | env116 | Environmental | *V. cholerae* Non-O1/ Non-O139 | May,2012 | 0 | 0 |
| 109 | env122A | Environmental | *V. cholerae* Non-O1/ Non-O139 | June,2012 | 0 | 0 |
| 110 | env124 | Environmental | *V. cholerae* Non-O1/ Non-O139 | June,2012 | 0 | 0 |
| 111 | env125 | Environmental | *V. cholerae* Non-O1/ Non-O139 | June,2012 | 0 | 0 |
| 112 | env127 | Environmental | *V. cholerae* Non-O1/ Non-O139 | June,2012 | 0 | 0 |
| 113 | env128 | Environmental | *V. cholerae* Non-O1/ Non-O139 | June,2012 | 0 | 42.4 |
| 114 | env133 | Environmental | *V. cholerae* Non-O1/ Non-O139 | June,2012 | 0 | 0 |
| 115 | env138 | Environmental | *V. cholerae* Non-O1/ Non-O139 | June,2012 | 0 | 43.2 |
| 116 | env140 | Environmental | *V. cholerae* Non-O1/ Non-O139 | June,2012 | 0 | 24.3 |
| 117 | env145 | Environmental | *V. cholerae* Non-O1/ Non-O139 | June,2012 | 0 | 70.0 |
| 118 | env146 | Environmental | *V. cholerae* Non-O1/ Non-O139 | June,2012 | 0 | 68.0 |
| 119 | env150 | Environmental | *V. cholerae* Non-O1/ Non-O139 | June,2012 | 0 | 0 |
| 120 | env155 | Environmental | *V. cholerae* Non-O1/ Non-O139 | June,2012 | 0 | 22.7 |
| 121 | env157 | Environmental | *V. cholerae* Non-O1/ Non-O139 | June,2012 | 0 | 7.1 |
| 122 | env160 | Environmental | *V. cholerae* Non-O1/ Non-O139 | June,2012 | 0 | 0 |
| 123 | env161 | Environmental | *V. cholerae* Non-O1/ Non-O139 | June,2012 | 0 | 0 |
| 124 | env164 | Environmental | *V. cholerae* Non-O1/ Non-O139 | June,2012 | 0 | 23.1 |
| 125* | env165 | Environmental | *V. cholerae* Non-O1/ Non-O139 | June,2012 | 4.3 | 33.3 |
| 126 | env166 | Environmental | *V. cholerae* Non-O1/ Non-O139 | June,2012 | 0 | 0 |
| 127 | env167 | Environmental | *V. cholerae* Non-O1/ Non-O139 | June,2012 | 0 | 0 |
| 128 | env180 | Environmental | *V. cholerae* Non-O1/ Non-O139 | July,2012 | 16.7 | 11.2 |
| 129 | env188 | Environmental | *V. cholerae* Non-O1/ Non-O139 | July,2012 | 0 | 0 |
| 130 | env189 | Environmental | *V. cholerae* Non-O1/ Non-O139 | July,2012 | 0 | 16.8 |
| 131 | env202 | Environmental | *V. cholerae* Non-O1/ Non-O139 | July,2012 | 0 | 29.2 |
| 132 | env205 | Environmental | *V. cholerae* Non-O1/ Non-O139 | July,2012 | 0 | 2.1 |
| 133 | env249 | Environmental | *V. cholerae* Non-O1/ Non-O139 | July,2012 | 0 | 0 |
| 134 | env269 | Environmental | *V. cholerae* Non-O1/ Non-O139 | July,2012 | 0 | 0 |
| 135 | env279 | Environmental | *V. cholerae* Non-O1/ Non-O139 | July,2012 | 0 | 0 |
| 136 | env287 | Environmental | *V. cholerae* Non-O1/ Non-O139 | July,2012 | 0 | 0 |
| 137 | env310 | Environmental | *V. cholerae* Non-O1/ Non-O139 | Aug,2012 | 0 | 11.4 |
| 138 | env311 | Environmental | *V. cholerae* Non-O1/ Non-O139 | Aug,2012 | 0 | 5.7 |
| 139 | env353 | Environmental | *V. cholerae* Non-O1/ Non-O139 | Sep,2012 | 0 | 0 |
| 140* | env357 | Environmental | *V. cholerae* Non-O1/ Non-O139 | Sep,2012 | 1.0 | 6.6 |
| 141 | env382 | Environmental | *V. cholerae* Non-O1/ Non-O139 | Sep,2012 | 0 | 6.0 |
| 142 | env385 | Environmental | *V. cholerae* Non-O1/ Non-O139 | Sep, 2012 | 0 | 12.5 |
| 143 | env397 | Environmental | *V. cholerae* Non-O1/ Non-O139 | Oct,2012 | 0 | 0 |
| 144 | env424 | Environmental | *V. cholerae* Non-O1/ Non-O139 | Oct,2012 | 0 | 37.9 |
| 145* | env485 | Environmental | *V. cholerae* Non-O1/ Non-O139 | Nov,2012 | 13.1 | 86.9 |
| 146 | env486 | Environmental | *V. cholerae* Non-O1/ Non-O139 | Nov,2012 | 0 | 0 |
| 147 | env512 | Environmental | *V. cholerae* Non-O1/ Non-O139 | Dec,2012 | 0 | 36.4 |
| 148 | env539 | Environmental | *V. cholerae* Non-O1/ Non-O139 | Dec,2012 | 30.2 | 1.8 |
| 149 | env571 | Environmental | *V. cholerae* Non-O1/ Non-O139 | Jan,2013 | 47.7 | 6.0 |
| 150 | env575 | Environmental | *V. cholerae* Non-O1/ Non-O139 | Jan,2013 | 0 | 0 |
| 151 | env598 | Environmental | *V. cholerae* Non-O1/ Non-O139 | Feb,2013 | 0 | 0 |
| 152 | env605 | Environmental | *V. cholerae* Non-O1/ Non-O139 | Feb,2013 | 0 | 0 |
| 153* | env625 | Environmental | *V. cholerae* Non-O1/ Non-O139 | Mar,2013 | 0 | 6.5 |
| 154 | env639 | Environmental | *V. cholerae* Non-O1/ Non-O139 | Mar,2013 | 0 | 0 |
| 155 | env715 | Environmental | *V. cholerae* Non-O1/ Non-O139 | Apr,2013 | 0 | 1.2 |
| 156 | env717 | Environmental | *V. cholerae* Non-O1/ Non-O139 | Apr,2013 | 0 | 0 |
| 157 | env721 | Environmental | *V. cholerae* Non-O1/ Non-O139 | May,2013 | 0 | 23.1 |
| 158 | env736 | Environmental | *V. cholerae* Non-O1/ Non-O139 | May,2013 | 0 | 0 |
| 159 | env913 | Environmental | *V. cholerae* Non-O1/ Non-O139 | June,2013 | 0 | 0 |
| 160 | env917 | Environmental | *V. cholerae* Non-O1/ Non-O139 | June,2013 | 0 | 2.2 |
| 161 | env975 | Environmental | *V. cholerae* Non-O1/ Non-O139 | July,2013 | 0 | 0 |
| 162 | env993 | Environmental | *V. cholerae* Non-O1/ Non-O139 | July,2013 | 0 | 5.2 |
| 163 | env1067 | Environmental | *V. cholerae* Non-O1/ Non-O139 | Aug,2013 | 0 | 0 |
| 164* | env1095 | Environmental | *V. cholerae* Non-O1/ Non-O139 | Aug,2013 | 93.0 | 73.7 |
| 165 | env1157 | Environmental | *V. cholerae* Non-O1/ Non-O139 | Sep,2013 | 0 | 0 |
| 166 | env1164 | Environmental | *V. cholerae* Non-O1/ Non-O139 | Sep,2013 | 0 | 0 |
| 167* | env1268 | Environmental | *V. cholerae* Non-O1/ Non-O139 | Oct,2013 | 0 | 22.8 |
| 168* | env1271 | Environmental | *V. cholerae* Non-O1/ Non-O139 | Oct,2013 | 0 | 1.9 |
| 169 | env1374 | Environmental | *V. cholerae* Non-O1/ Non-O139 | Nov,2013 | 0 | 0 |
| 170 | env1378 | Environmental | *V. cholerae* Non-O1/ Non-O139 | Nov,2013 | 0 | 0 |
| 171 | env1404 | Environmental | *V. cholerae* Non-O1/ Non-O139 | Dec,2013 | 0 | 0 |
| 172 | env1444 | Environmental | *V. cholerae* Non-O1/ Non-O139 | Dec,2013 | 0 | 28.6 |
| 173 | N16961 | Reference | *V. cholerae* O1 | - | 30.0 | 80.0 |
| 174 | O395 | Reference | *V. cholerae* O1 | - | 0 | 0 |

*indicates the strains that converted to rugose after 24 hours
